# Identification of Hypoxia Induced Metabolism Associated Genes in Pulmonary Hypertension

**DOI:** 10.3389/fphar.2021.753727

**Published:** 2021-11-05

**Authors:** Yang-Yang He, Xin-Mei Xie, Hong-Da Zhang, Jue Ye, Selin Gencer, Emiel P. C. van der Vorst, Yvonne Döring, Christian Weber, Xiao-Bin Pang, Zhi-Cheng Jing, Yi Yan, Zhi-Yan Han

**Affiliations:** ^1^ School of Pharmacy, Henan University, Kaifeng, China; ^2^ State Key Laboratory of Cardiovascular Disease and FuWai Hospital, Chinese Academy of Medical Sciences and Peking Union Medical College, Beijing, China; ^3^ Institute for Cardiovascular Prevention (IPEK), Ludwig-Maximilians-University Munich, Munich, Germany; ^4^ DZHK (German Centre for Cardiovascular Research), Partner Site Munich Heart Alliance, Munich, Germany; ^5^ Interdisciplinary Center for Clinical Research (IZKF), RWTH Aachen University, Aachen, Germany; ^6^ Institute for Molecular Cardiovascular Research (IMCAR), RWTH Aachen University, Aachen, Germany; ^7^ Department of Pathology, Cardiovascular Research Institute Maastricht (CARIM), Maastricht University Medical Centre, Maastricht, Netherlands; ^8^ Department of Angiology, Swiss Cardiovascular Center, Inselspital, Bern University Hospital, University of Bern, Bern, Switzerland; ^9^ Department of Biochemistry, Cardiovascular Research Institute Maastricht (CARIM), Maastricht University Medical Centre, Maastricht, Netherlands; ^10^ Munich Cluster for Systems Neurology (SyNergy), Munich, Germany; ^11^ State Key Laboratory of Complex, Severe, and Rare Diseases, Department of Cardiology, Peking Union Medical College Hospital, Chinese Academy of Medical Sciences and Peking Union Medical College, Beijing, China

**Keywords:** pulmonary hypertension, hypoxia, metabolism associated genes, metabolomics, transcriptomics

## Abstract

**Objective:** Pulmonary hypertension (PH) associated with hypoxia and lung disease (Group 3) is the second most common form of PH and associated with increased morbidity and mortality. This study was aimed to identify hypoxia induced metabolism associated genes (MAGs) for better understanding of hypoxic PH.

**Methods:** Rat pulmonary arterial smooth muscle cells (PASMCs) were isolated and cultured in normoxic or hypoxic condition for 24 h. Cells were harvested for liquid chromatography-mass spectrometry analysis. Functional annotation of distinguishing metabolites was performed using Metaboanalyst. Top 10 enriched metabolite sets were selected for the identification of metabolism associated genes (MAGs) with a relevance score >8 in Genecards. Transcriptomic data from lungs of hypoxic PH in mice/rats or of PH patients were accessed from Gene Expression Omnibus (GEO) database or open-access online platform. Connectivity Map analysis was performed to identify potential compounds to reverse the metabolism associated gene profile under hypoxia stress. The construction and module analysis of the protein-protein interaction (PPI) network was performed. Hub genes were then identified and used to generate LASSO model to determine its accuracy to predict occurrence of PH.

**Results:** A total of 36 altered metabolites and 1,259 unique MAGs were identified in rat PASMCs under hypoxia. 38 differentially expressed MAGs in mouse lungs of hypoxic PH were revealed, with enrichment in multi-pathways including regulation of glucose metabolic process, which might be reversed by drugs such as blebbistatin. 5 differentially expressed MAGs were displayed in SMCs of Sugen 5416/hypoxia induced PH rats at the single cell resolution. Furthermore, 6 hub genes (Cat, Ephx1, Gpx3, Gstm4, Gstm5, and Gsto1) out of 42 unique hypoxia induced MAGs were identified. Higher Cat, Ephx1 and lower Gsto1 were displayed in mouse lungs under hypoxia (all p < 0.05), in consistent with the alteration in lungs of PH patients. The hub gene-based LASSO model can predict the occurrence of PH (AUC = 0.90).

**Conclusion:** Our findings revealed six hypoxia-induced metabolism associated hub genes, and shed some light on the molecular mechanism and therapeutic targets in hypoxic PH.

## Background

Pulmonary hypertension (PH) is a heterogenous disorder and characterized by high pulmonary artery pressure and elevated pulmonary vascular resistance that usually leads to right heart failure and even death (Hoeper et al., 2013). The pathogenesis of PH is driven or initiated by multiple factors including genetic mutations, epigenetic alterations, inflammation, altered metabolism, and environment insults such as hypoxia, drugs and toxins (de Jesus Perez, 2016). PH associated with hypoxia and lung disease (Group 3 PH) is the second most common form of PH and is associated with increased morbidity and mortality (Strange et al., 2012; McGettrick and Peacock, 2020). However, most of PAH-specific medications including endothelin receptor antagonists, phosphodiesterase type 5 inhibitors, and prostacyclin have not shown consistent benefit in the Group 3 PH (Harder and Waxman, 2020). Hence, the discovery of some targets for hypoxic PH would be of great value for those Group 3 population.

On one hand, hypoxic PH share a similar pathological changes with other type of PH including the accumulation of pulmonary artery adventitial fibroblasts and excessive deposition of extracellular matrix proteins, proliferation and migration of pulmonary arterial smooth muscle cells (PASMCs) and endothelial cell proliferation that promotes intimal thickening (Meyrick and Reid, 1980; Stenmark et al., 2006; Chen et al., 2009). Growing evidence indicates that the excessive proliferation, apoptosis resistance and a contractile-to-synthetic phenotype switch of PASMCs has emerged as essential mechanisms in the remodeling of the pulmonary vasculature associated with hypoxic PH. For example, our previous study demonstrated that overexpression of DNMT3B (encoding DNA methyltransferase 3B) mitigated the remodeling of hypoxic PH in mice, suggesting the protective role of DNMT3B on the pulmonary vascular remodeling via the modulation of PASMC proliferation/migration (Yan et al., 2020). In addition, the markedly increased CD146 expression in PASMCs under hypoxia and hypoxia-inducible transcription factor 1 alpha (HIF-1α) transcriptional program reinforce each other to enable PASMCs to adopt a more synthetic phenotype. Genetic ablation of CD146 in smooth muscle cells mitigates pulmonary vascular remodeling in chronic hypoxic mice (Luo et al., 2019). On the other hand, the activation of transcription factor HIF-1α under hypoxia stress induces the expression of glycolytic genes and actively suppressed mitochondrial oxidative phosphorylation by inducing pyruvate dehydrogenase kinase 1, which hampers pyruvate dehydrogenase from using pyruvate to fuel tricarboxylic acid cycle (Papandreou et al., 2006). The overall picture of metabolic shift and metabolite perturbations in PH have become clearer in recent years due to the application of metabolomics. The interventions of specific enzymes to restore the abnormal metabolism open a new avenue for the treatment of PH. For example, inhibitor of glucose-6-phosphate dehydrogenase, a rate-limiting enzyme in pentose phosphate pathway, was reported to modulate DNA methylation and alleviate pulmonary artery remodeling (Kitagawa et al., 2021). Sphingosine kinases 1 (SphK1) is determinant for the synthesis of sphingosine-1-phosphate, an critical mediator for cell proliferation, migration and angiogenesis. Deficiency and pharmacological inhibition of SphK1 mitigated the development of hypoxic PH *in vivo* and SphK1 deficiency inhibited PASMC proliferation *in vitro* (Chen et al., 2014).

Here, we sought to examine the metabolic profiles in PASMCs and metabolism associated genes in response to hypoxia. In combination of transcriptomics data sets from both PH rodent models and PH patients, we aimed to identify hypoxia induced metabolism associated hub genes, which might be prospective targets for the treatment of hypoxic PH.

## Methods

### Rat PASMCs Isolation and Cell Culture

Rat PASMCs were isolated as previously described (Yan et al., 2020). Briefly, 6-week old Sprague-Dawley rat purchased from Charles River (Beijing, China) was sacrificed, which was approved by the Ethics and Animal Care and Use Committee of Henan University. The lungs were excised and rinsed with phosphate-buffered saline (PBS). The pulmonary arteries were isolated followed by removal of the adventitia under the microscope. Minced arteries were attached to bottom of Petri dish and then immersed by Dulbecco’s modified eagle medium/F12 containing 20% fetal bovine serum, 100 U/mL penicillin and 100 μg/ml streptomycin in a 37°C, 5% CO2 humidified incubator. Three days later, non-adherent cells were removed, and the adherent cells that had grown to 90% confluence were considered as passage 0 PASMCs. Passages 3 were used for the subsequent hypoxia experiments.

### Hypoxia Experiment

Rat PASMCs at a density of 1 × 10^5^/ml were seeded into 6-well plates and then placed in a 37°C, 5% CO2 humidified incubator. 24 h later cells were starved with Dulbecco’s modified eagle medium/F12 containing 0.5% fetal bovine serum. Medium were refreshed and cells were then either cultured in normoxia condition (21% O_2_) or hypoxia condition (1%O_2_) in the incubator 24 h post-starvation. 24 h later cells under normaxia or hypoxia (n = 6 per group) were collected respectively. Cells were washed and centrifuged at 300 g at 4°C for 10 min and resuspend with 1 ml PBS. Cells were then counted with erythrocytometers and diluted to a final density of 1 × 10^5^/ml with PBS. 1 ml cell suspension per sample was transferred to 1.5 ml clean tube and centrifugated at 300 g at 4°C for 5 min. Supernatants were discarded and cell pellets were stored at −80°C before further use. No repeated freezing and thawing occurred before sample processing to avoid potential degradation risks of metabolites.

### Sample Preparation

1 ml of MeOH:ACN:H2O (2:2:1, v/v) solvent mixture was added into the cell samples. The cell mixtures were vortexed for 30 s followed by sonification at 4°C in the water bath for 10 min. The mixtures were transferred to liquid nitrogen for 1 min and thawed at room temperature followed by sonification at 4°C in the water bath for 10 min. After the repeat for 3 times, mixtures were put at −20°C for 1 h to facilitate protein precipitation. Mixtures were centrifuged at 13,000 rpm at 4°C for 15 min, supernatants were discarded, and pellets were vacuum dried and reconstituted with 100 μL of ACN:H2O (1:1, v/v). Mixtures were vortexed for 30 s and sonicated as previously stated followed by centrifugation at 13,000 rpm at 4°C for 15 min. Supernatants were stored at −80°C prior to Liquid chromatography-mass spectrometry (LC/MS) analysis.

### Liquid Chromatography-Mass Spectrometry Analysis

Liquid chromatographic separation for processed cell samples was achieved on a ZORBAX Eclipse Plus C18 column (2.1 × 100 mm, 3.5 μm, Agilent, United States) maintained at 45°C, whereas mass spectrometry was performed on a Nexera X2 system (Shimadzu, Japan) coupled with a Triple TOF 5600 quadrupole-time-of-flight mass spectrometer (AB SCIEX, United States). The temperature of the sample chamber was maintained at 7°C. The gradient elution steps were shown in Supplementary Table S1. The injected sample volume was 10 μL for each run in the full loop injection mode, and the flow rate of the mobile phase was 0.5 ml/min. The mobile phase A was mainly composed of water and contains 0.1% formic acid. The mobile phase B was mainly composed of acetonitrile and contains 0.1% formic acid. Water (LC-MS grade) and acetonitrile (LC-MS grade) were purchased from Fisher Scientific. The purity of formic acid purchased from Acros was greater than 98%.

### Data Availability and Process

In terms of metabolomics study, data preprocessing was performed before pattern recognition. The original data was processed by the instrument’s own metabolomics processing software Progenesis QI (Waters Corporation, Milford, United States) for baseline filtering, peak identification, integration, retention time correction, peak alignment, and normalization. Finally, a data matrix of retention time, mass-to-charge ratio and peak intensity were obtained. The integrated data matrix was imported into the SIMCA-P+ (v 14.1) software package (Umetrics, Umeå, and Sweden), and partial least squares discriminant analysis (PLS-DA) was used to distinguish the overall difference in metabolic profile between groups. In PLS-DA analysis, variables with a variable weight value (Variable Important in Projection, VIP) > 1 were considered to be distinguishing among groups.

With regard to transcriptomic analysis, we accessed the data set GSE 1909 (Gharib et al., 2005) based on GPL1674 including mouse lung tissues during development of hypoxia-induced PH (days 1–21) and resolution of PH after return to normoxia (days 22–35). Mice were sampled during nine time-points and there were 4 replicates at each time-point. We selected samples at day 1, day 14, day 21, and day 35 after hypoxia for further analysis. Differential expression analysis was performed using the limma package in R (v3.6.3.). (Ritchie et al., 2015). *p* value <0.05 and fold change (FC) of gene expression >1.5 or <0.67 between two groups were considered to be differentially expressed genes (DEGs). To validate the hub gene in lung tissues from PH patients, we selected the datasets with a relative large sample size (lung tissues from control subjects >20, and lung tissues from PH patients >50) and two datasets GSE117261 (Stearman et al., 2019) consisting of 25 control subjects and 58 PH patients, and GSE24988 (Mura et al., 2012) including 22 controls and 62 PH patients were included for further analysis. Both were based on GPL6244. The ComBat function in the sva package was used to merge these two data sets and remove the batch effect (Leek et al., 2012). In addition, single cell RNA sequencing data from lung tissues of monocrotaline, Sugen 5416/hypxoixa (SuHx) or control rats were downloaded from an open-access online platform (http://mergeomics.research.idre.ucla.edu/PVDSingleCell/). (Hong et al., 2020)

### Connectivity Map Analysis

The Connectivity Map (CMap) (https://portals.broadinstitute.org/cmap) is an open resource that connect genes, small molecules, and disease by virtue of common gene-expression signatures (Lamb et al., 2006). Drugs with Mean < −0.4 and *p* < 0.05 in CMap analysis were considered to be potential small molecular compounds that can reverse altered expression of user-defined gene list in cell lines.

### Identification of Hub Genes and Network Interaction Visualization

Top 10 enriched metabolite pathways were identified by enrichment analysis of metabolite sets with online open-access platform Metaboanalyst (Chong et al., 2019) (v5.0, https://www.metaboanalyst.ca). Metabolism associated genes (MAGs) were acquired from Genecards (Safran et al., 2010) (https://www.genecards.org) database. The terms of top 10 enriched metabolite pathways were used as key word for analysis, and genes with relevance score >8 were regarded as MAGs. The intersection of MAGs and DEGs in mouse lung tissues of hypoxia induced PH was listed as gene set 1. The intersection of MAGs and DEGs in smooth muscle cells of SuHx versus control rats at single cell level was listed as gene set 2. The union set of these two gene sets were regarded as hypoxia induced metabolism associated genes. Protein protein interaction were analyzed and visualized by online tool STRING (v11.0, https://string-db.org). (Szklarczyk et al., 2019) Hub genes were identified by cytoHubba plugin with MCC algorithm or by MCODE plugin in Cytoscape (v 3.8.2) (Shannon et al., 2003).

### Construction of LASSO Regression Model and Receiver Operating Characteristic Curve Analysis

To distinguish PH patients from control subjects, Least Absolute Shrinkage and Selection Operator (LASSO) regression model was constructed with the expression profiles of 6 metabolism associated hub genes by glmnet package in R. A model index for each sample was generated using the regression coefficients from the LASSO analysis to weight the expression value of the selected genes. GSE117261 data set were randomly assigned to training set (70%) and test set (30%). ROC curves were generated to evaluate the ability of LASSO model to identify PH by ROCR package.

### Data Visualization and Statistics

The sample distribution of hypoxia exposed rat PASMCs (Hx) and control PASMCs (Nor), or mouse lung tissues under hypoxia at the indicated days were visualized in scatter plot with R package ggplot2. The distribution of data sets GSE117261 and GSE24988 before and after removal of batch effect was also visualized in scatter plot with ggplot2 package. Distinct metabolites/genes between groups were visualized in volcano plot. Heatmap was generated to visualize the expression of all the distinct metabolites or indicated genes in each individual sample using pheatmap package in R. Venn diagram was generated to visualize the overlap of the indicated gene sets using R package VennDiagram. Pathway enrichment of indicated genes were analyzed in functional annotation bioinformatics tool DAVID (Huang et al., 2009) (v6.8, https://david.ncifcrf.gov/tools.jsp) and were then plotted with barplot function in R. Uniform manifold approximation and projection (UMAP) was generated based on Seurat_umap.coords data to visualize each clusters under different conditions. Hub gene network was visulized by Cytoscape.

Data are presented as the mean ± standard error of the mean (SEM). When two groups were compared, statistical differences were assessed with unpaired 2-tailed unpaired *t*-test if normally distributed. Otherwise, Mann-Whitney *U* test was utilized. Comparisons of more than three groups were performed by analysis of variance (ANOVA) and Tukey’s post-hoc test or Kruskal-Wallis test, as appropriate (GraphPad Prism 8). A two-sided *p* value of ≤0.05 was used to determine significant differences among groups.

## Results

### Altered Metabolite Profiles in Rat PASMCs in Response to Hypoxia and Identification of Metabolism Associated Genes

**FIGURE 1:**
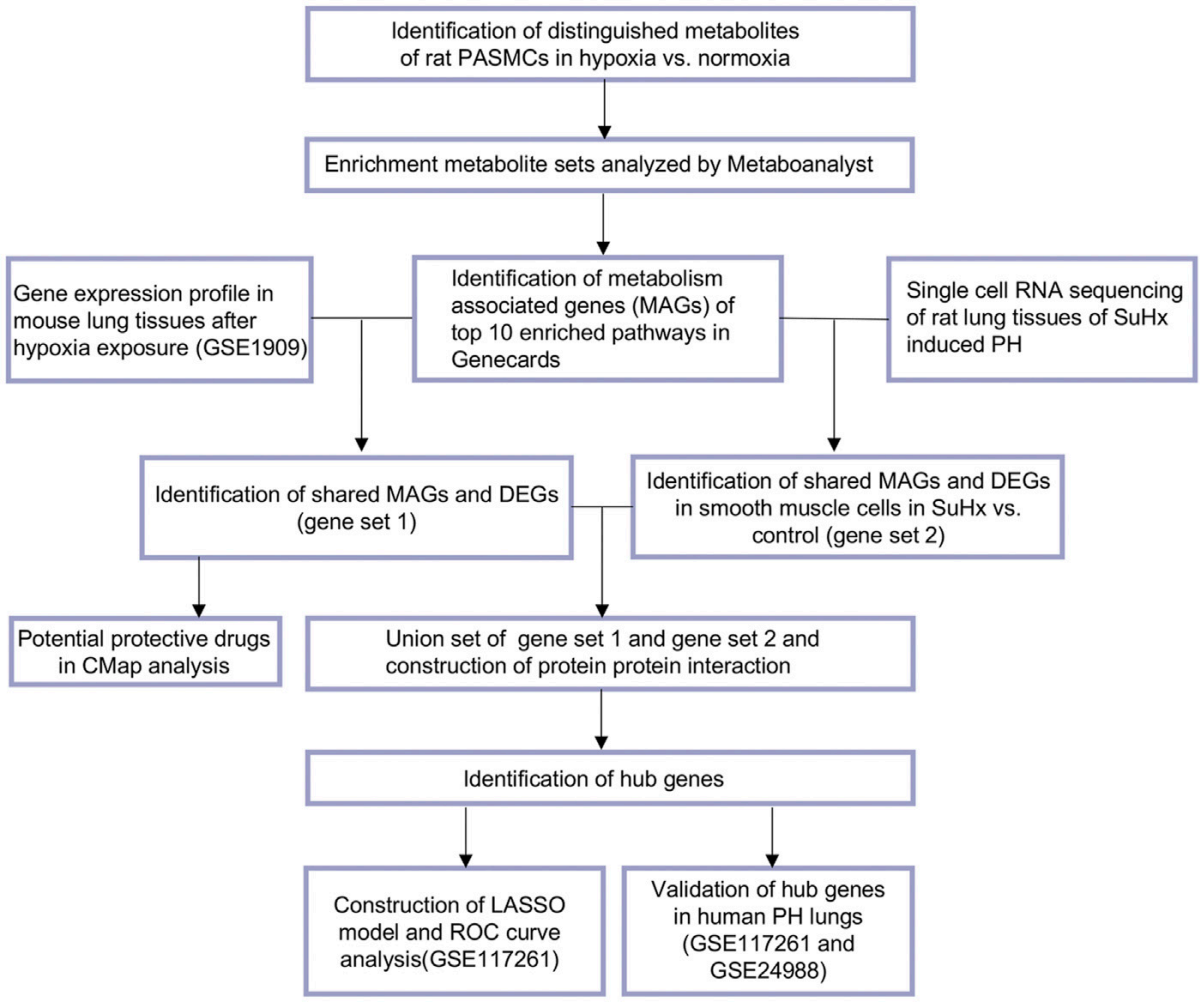
Main analysis flowchart. Identification of distinguishing metabolites of rat pulmonary arterial smooth muscle cells (PASMCs) after hypoxia (1% O_2_) exposure for 24 h versus PASMCs in normoxia condition. Enrichment metabolite sets were analyzed by Metaboanalyst (v 5.0). Top 10 enriched pathways were revealed, and 1,259 metabolism associated genes (MAGs) with a relevance score ≥8 of top 10 enriched pathways in Genecards were identified. Next the shared MAGs and differentially expressed genes (DEGs) in mouse lung tissues of hypoxia induced pulmonary hypertension (PH) were identified (gene set 1), and potential small molecular compounds (drugs) to reverse the altered expression of gene set 1 were revealed by Connectivity Map (CMap) analysis. Dataset of single cell RNA sequencing of rat lung tissues of Sugen 5416/hypoxia (SuHx) induced PH were obtained and shared genes of MAGs and DEGs in smooth muscle cells of SuHx versus control rats were listed in gene set 2. Gene set 1 and gene set 2 were combined and protein protein interaction were constructed and visualized. Hub gene were identified by cytoHubba or MCODE add-ins in Cytoscape. LASSO model was constructed with the expression profiles of hub genes and ROC curves generated to evaluate the ability of LASSO model to identify PH in dataset from GSE117261. Common hub genes were validated in human PH lung tissues versus controls in dataset from GSE117261 or combined datasets from GSE117261 and GSE24988.

**FIGURE 2:**
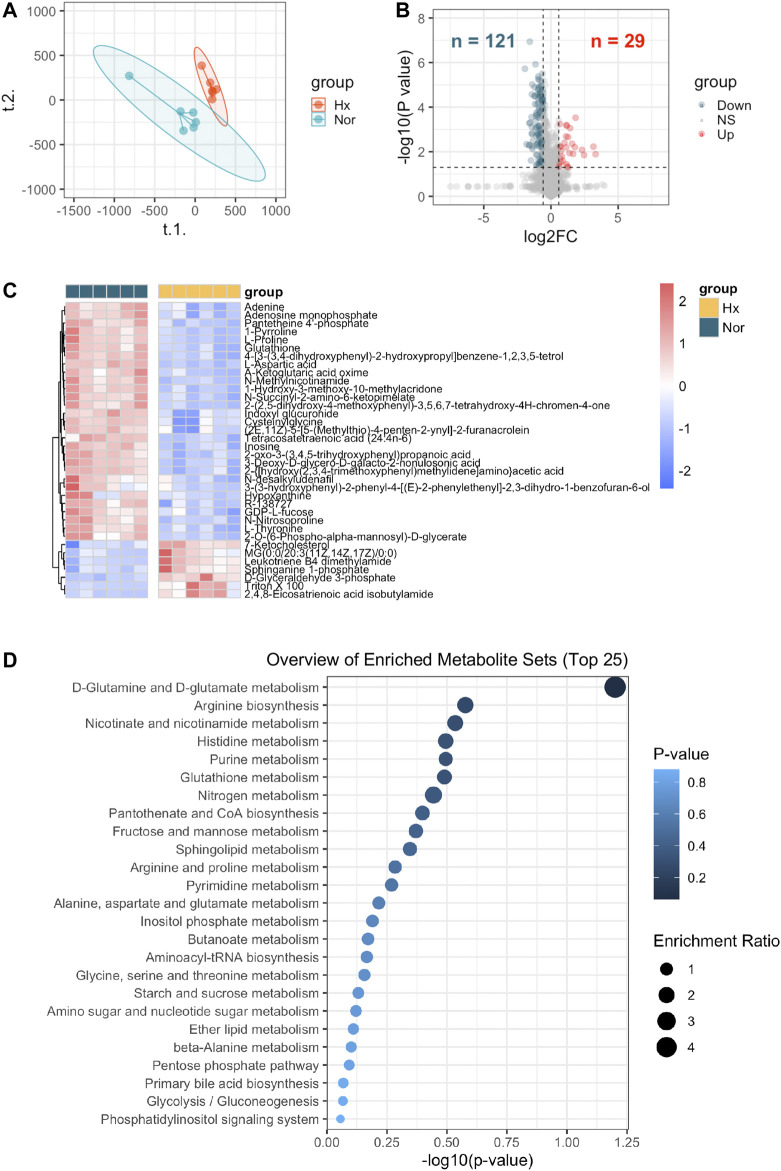
Identification of metabolites distinguishing rat PASMCs under hypoxia from normoxic controls and enriched metabolite sets. **(A)** Partial least squares discriminant analysis (PLS-DA) demonstrated a well separated sample distribution of rat PASMCs under hypoxia (Hx) and control PASMCs (Nor) for 24 h and visualized in scatter plot (n = 6). **(B)** 29 upregulated metabolites (red dots) and 121 down-regulated metabolites (dark green) were identified and visualized in volcano plot (Fold change >1.5 or <0.67 and *p* < 0.05). **(C)** Expression of distinguishing metabolites in **(B)** with VIP score >1 were visualized in heatmap. **(D)** Top 25 enrichment metabolite sets of distinguishing metabolites were identified in Metaboanalyst (v 5.0).

According to flow chart as illustrated in Figure 1, the metabolite profiles in rat PASMCs under hypoxia stress and control PASMCs were explored. We found that distribution of hypoxia exposed rat PASMCs differed from control cells by PLS-DA analysis (Figure 2A). 29 up-regulated and 121 down-regulated metabolites were observed in hypoxia challenged PASMCs compared to control cells (FC > 1.5 or <0.67, *p* < 0.05) (Figure 2B). Among those only 36 metabolites were identified to have a VIP score >1 and their relative expressions among each individual sample were visualized in heatmap, of which 7 were upregulated and 29 were downregulated in response to hypoxia (Figure 2C). Next, we demonstrated that the metabolites distinguishing hypoxic and normoxic rat PASMCs were enriched in metabolite sets such as D-Glutamine and D-glutamate metabolism, arginine biosynthesis, nicotinate, and nicotinamide metabolism etc., and the top 25 enriched metabolite sets were display in Figure 2D.

We next selected top 10 metabolite sets to determine the MAGs potentially in response to hypoxia. A total of 1,259 unique MAGs with a relevance score >8 was identified from GeneCards website (Supplementary Table S2).

### Identification of Differentially Expressed MAGs in Mouse Lungs of Hypoxia Induced PH and Potential Protective Drugs

**FIGURE 3:**
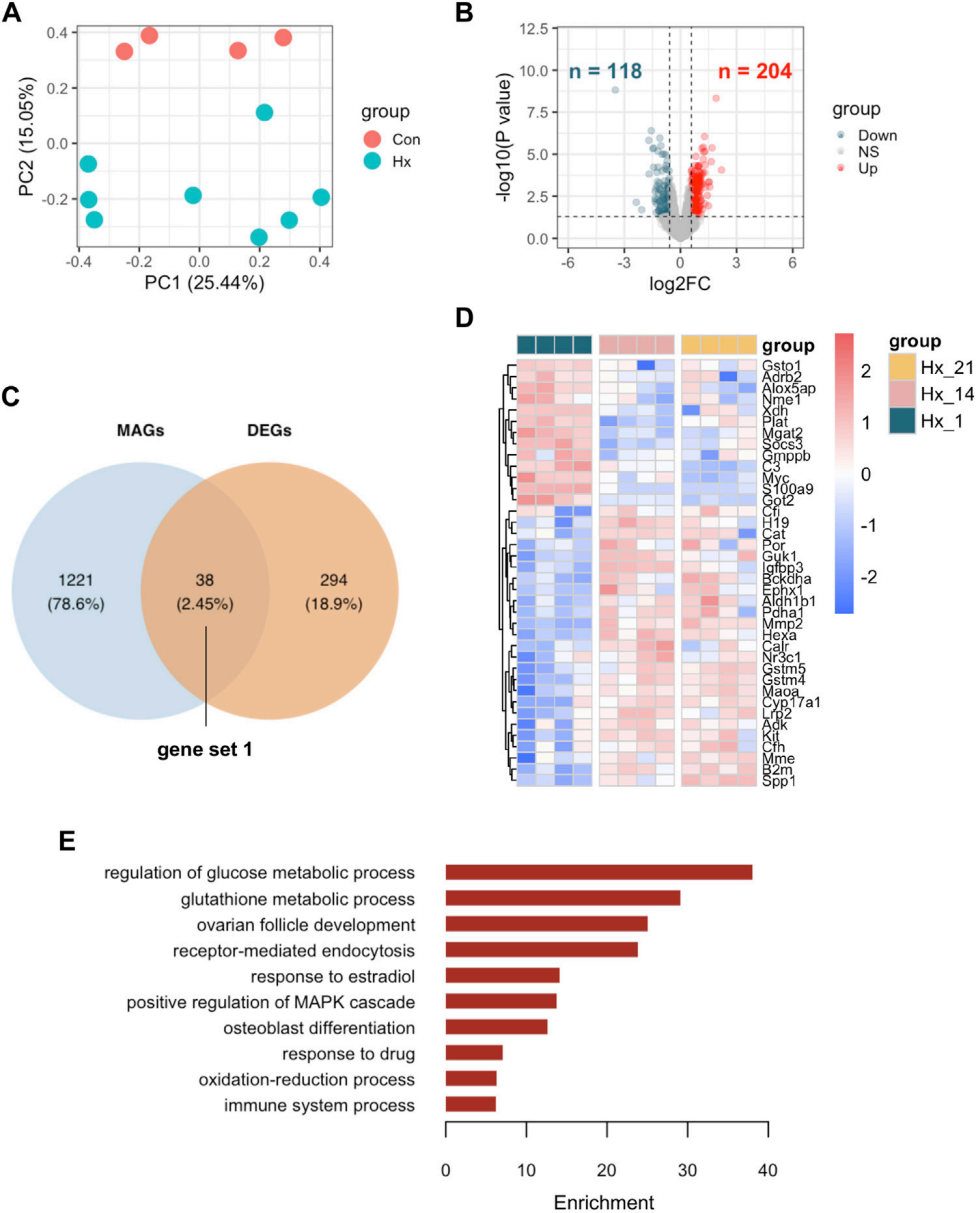
Identification of metabolism associated DEGs in mouse lungs of hypoxia induced PH and enriched pathways. **(A)** Principal component analysis (PCA) demonstrated a well separated sample distribution of mouse lung tissues of hypoxia at day 14 and at day 21 (Hx) and those of hypoxia at day 1 (Con) from GSE 1909 (n = 4 per condition). **(B)** 204 upregulated genes (red dots) and 118 down-regulated metabolites (dark green) were identified and visualized in volcano plot (Fold change >1.5 or <0.67 and *p* < 0.05).**(C)** Overlap of metabolism associated genes (MAGs) and DEGs between Hx and Con were visualized in Venn diagram; 38 overlapped genes was listed as gene set 1. **(D)** Expression of 38 overlapped genes were visualized in heatmap at indicated time point after hypoxia; Hx_21: day 21 after hypoxia; Hx_14: day 14 after hypoxia; Hx_1: day 1 after hypoxia. **(E)** The enriched GO ontology (biological process) were identified by functional annotation tool DAVID and visualized in bar plot.

To explore the differentially expressed MAGs in mouse lung tissues of hypoxia induced PH compared to control mice, we first scrutinize the DEGs in lung tissues of mice after hypoxia for 14 days or 21 days (Hx) compared to those after hypoxia for 1 day (Con) from GEO data set GSE 1909. Hx samples were well separate from Con samples (Figure 3A). In addition, 204 up-regulated and 118 down-regulated genes were displayed in Hx samples compared to Con samples (Figure 3B). The intersection of DEGs and MAGs is visualized in a Venn diagram (Figure 3C). The relative expression of 38 differentially expressed MAGs (gene set 1) in each individual lung tissue under hypoxia exposure at different time point were shown in heatmap, of which 25 were increased and 13 were decreased in Hx samples (Figure 3D). Next, the 38 MAGs were selected for functional analysis of GO ontology (biological process). It turned out that pathways including regulation of glucose metabolic process, glutathione metabolic process, receptor-mediated endocytosis, response to estradiol, positive regulation of MAPK cascade, etc. were enriched in response to chronic hypoxia exposure. In addition, CMap analysis were used to search for potential small molecular compounds to reverse altered expression of the 38 MAGs in gene set 1, and the top 10 most potential protective drugs including blebbistatin, corynanthine and butyl hydroxybenzoate were listed in Table 1.

**TABLE 1:** List of 10 most significant small molecular compounds provided by CMap analysis to reverse altered expression of 38 differentially expressed MAGs of gene set 1 in cell lines.

CMap name	Mean	Enrichment	*p*	Percent non-null
blebbistatin	−0.658	−0.89	0.02388	100
corynanthine	−0.578	−0.933	0.0005	100
butyl hydroxybenzoate	−0.547	−0.847	0.00024	100
canadine	−0.545	−0.84	0.00117	100
ciclosporin	−0.476	−0.639	0.00638	66
flurbiprofen	−0.476	−0.666	0.00965	80
dyclonine	−0.445	−0.73	0.0107	75
ramifenazone	−0.445	−0.674	0.02528	75
ketorolac	−0.437	−0.746	0.00822	75
trimethylcolchicinic acid	−0.423	−0.653	0.03416	75

### Identification of Metabolism Associated Differentially Expressed Genes in Smooth Muscle Cells of Sugen 5416/Hypoxia Induced PH Rats and the Expressions in Mouse Hypoxic Lung Tissues

**FIGURE 4:**
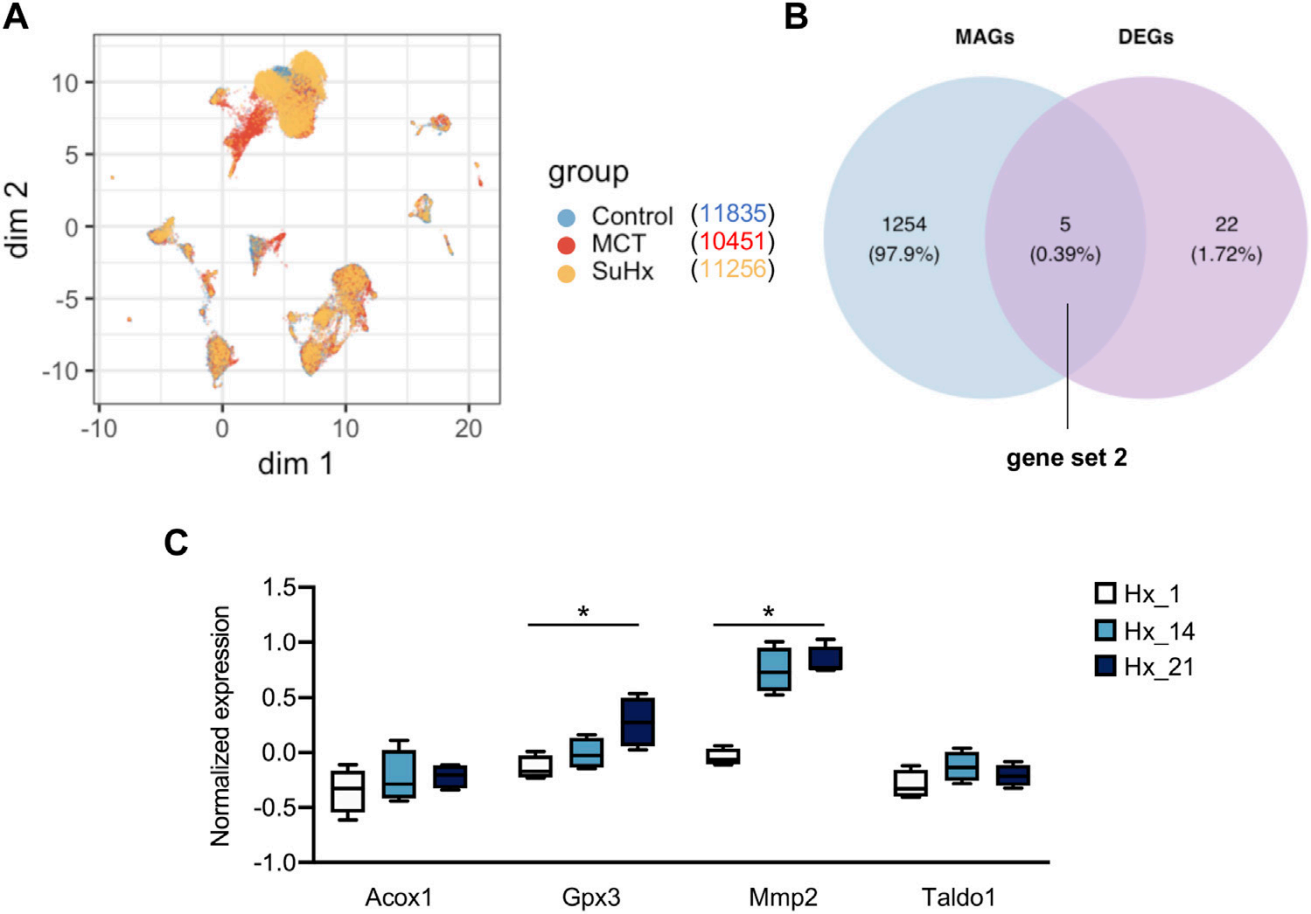
Identification of metabolism associated DEGs in SMC of SuHx rats and expressions in mouse lungs. **(A)** Uniform manifold approximation and projection (UMAP) was generated based on Seurat_umap.coords data from an open-access online platform (http://mergeomics.research.idre.ucla.edu/PVDSingleCell/). **(B)** Overlap of metabolism associated genes (MAGs) and DEGs in smooth muscle cells between Sugen 5416/hypoxia induced PH (SuHx) and control rats were visualized in Venn diagram; 5 overlapped genes was listed as gene set 2. **(C)** Expression of 4 overlapped genes (Aldh6a1 was not detected in lung tissues from GSE1909 dataset) were visualized in box plot at indicated time point after hypoxia; Hx_1: day 1 after hypoxia, Hx_14: day 14 after hypoxia, Hx_21: day 21 after hypoxia. Data represent mean ± SEM. *p < 0.05, as analyzed by one-way ANOVA with Tukey’s multiple comparisons or Kruskal-Wallis test respectively, as appropriate.

Due to the application of single cell RNA sequencing, we are allowed to examine the DEGs at a single cell resolution. Hence, we sought to further test the metabolism associated DEGs in smooth muscle cell from lung tissues of SuHx rats relative to control rats. As illustrated in Figure 4A, UMAP was generated based on Seurat_umap.coords data from an open-access online platform (http://mergeomics.research.idre.ucla.edu/PVDSingleCell/) and displayed the overlap of distinct clusters from control, monocrotaline or SuHx lungs. Overlap of 1,259 MAGs and 27 repoeted DEGs in smooth muscle cells (Supplementary Table S3) between SuHx and control rats were visualized in Venn diagram, with 5 overlapped genes (*Acox1*, *Gpx3*, *Mmp2*, *Taldo1,* and *Aldh6a1*) listed as gene set 2 (Figure 4B). We next examined the expression of 5 differentially expressed MAGs in lung tissues of hypoxia induced PH mice. All genes except *Aldh6a1* were detected in lung tissues from GSE1909 data set. *Mmp2* and *Gpx3* were significantly elevated in mouse lung tissues after hypoxia exposure for 21 days (*p* < 0.05), and had a tendancy towards elevation at day 14 post-hypoxia even though didn’t reach any significance (Figure 4C).

### Identification of Hypoxia Induced Metabolism Associated Hub Genes and the Expressions in Hypoxic Lung Tissues

**FIGURE 5:**
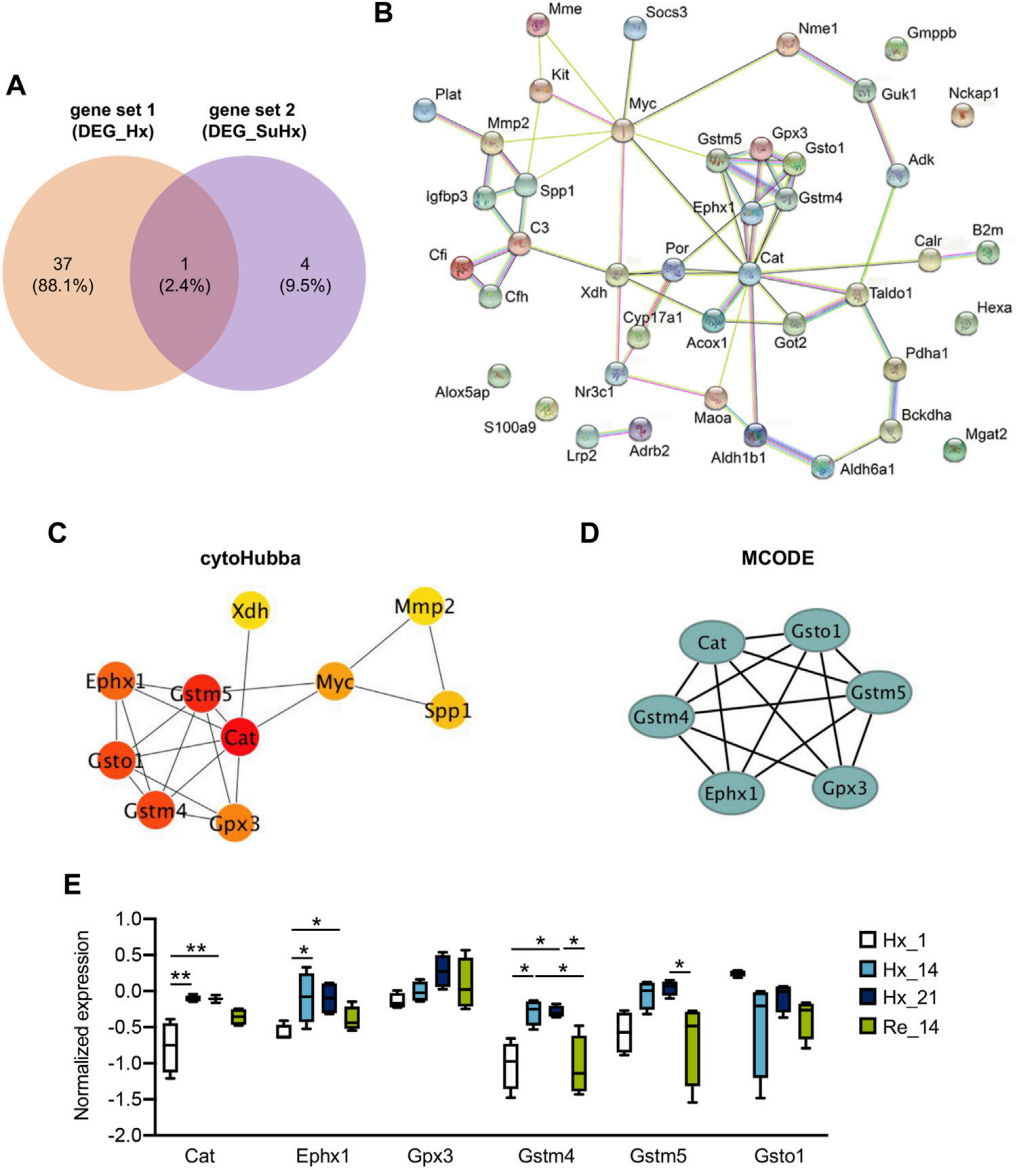
Identification of hypoxia induced metabolism associated hub genes and the expressions in hypoxic lungs. **(A)** Overlap of gene set 1 and gene set 2 were visualized in Venn diagram; 42 genes in union set of both gene sets were regarded as hypoxia induced metabolism associated genes. **(B)** Protein protein interaction among the 42 genes were analyzed and visualized by online tool STRING (v11.0). **(C)** Identification of 10 hub genes by cytoHubba plugin with MCC algorithm in Cytoscape (v 3.8.2); each circle represents unique gene and the redder the color is, the higher the MCC score is. **(D)** Identification of 6 hub genes by MCODE plugin in Cytoscape (v 3.8.2); each circle represents unique gene. **(E)** Expression of 6 shared hub genes were visualized in box plot at indicated time point after hypoxia or recovery to normoxia; Hx_1: day 1 after hypoxia, Hx_14: day 14 after hypoxia, Hx_21: day 21 after hypoxia, Re_14: day 14 of normoxia recovery after 21 days of hypoxia exposure. Data represent mean ± SEM. *p < 0.05, **p < 0.01, as analyzed by one-way ANOVA with Tukey’s multiple comparisons or Kruskal-Wallis test respectively, as appropriate.

We next combined the differentially expressed MAGs in mouse lungs of hypoxia induced PH (gene set 1) and differentially expressed MAGs in smooth muscle cells of lungs from SuHx rats (gene set 2). A total of 42 genes of both gene sets were then potentially regarded as hypoxia induced MAGs (Figure 5A). 61 Protein protein interactions among the 42 genes were analyzed and visualized by STRING (v11.0) (Figure 5B). To identify the hub genes of the protein protein network, cytoHubba plugin with MCC algorithm and MCODE plugin in Cytoscape were utilized, respectively. Ten hub genes were discovered by cytoHubba (Figure 5C) and six hub genes were identified by MCODE (Figure 5D). Of note, six genes including *Cat*, *Ephx1*, *Gpx3*, *Gstm4*, *Gstm5,* and *Gsto1* were identified as core hub genes as they were unveiled by both methods. We next investigated the expressions of six hub genes in mouse lung tissues from GSE1909 data set at indicated time point after hypoxia or recovery to normoxia after hypoxia exposure. *Cat* encoding catalase, *Ephx1* encoding epoxide hydrolase 1 and *Gstm4* encoding glutathione S-transferase, mu 4 were increased both at day 14 and day 21 after hypoxia stress compared to control group (all *p* < 0.05) and declined after 14 days recovery to normoxia, although the reduction is only significant in *Gstm4* (*p* < 0.05). A similar trend was also observed in *Gpx3* encoding glutathione peroxidase 3 and *Gstm5* encoding glutathione S-transferase mu 5. In contrast, *Gsto1* encoding glutathione S-transferase omega 1 were declined at day 14 and day 21 after hypoxia stress and didn’t alter after recovery to ambient atmosphere (Figure 5E).

### Evaluation of Hypoxia Induced Metabolism Associated Hub Genes in Lung Tissues of Patients With Pulmonary Hypertension

**FIGURE 6:**
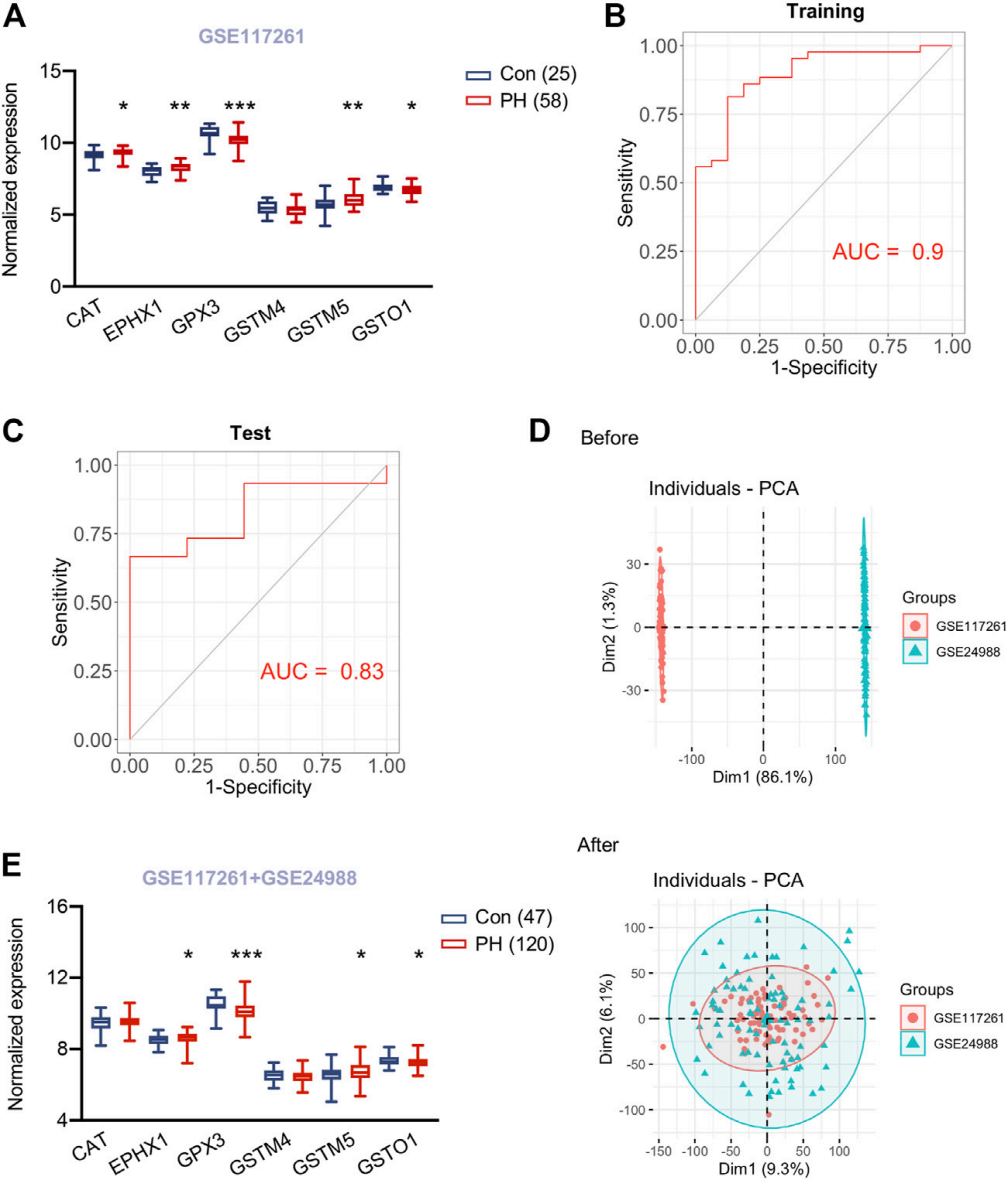
Validation of hub genes in lungs of PH patients and a predicting model for PH. **(A)** Expression of six hypoxia induced metabolism associated hub genes in lung tissues of 58 patients with pulmonary hypertension (PH) and 25 control subjects from GSE117261. **(B)** ROC curve analysis of training set (GSE117261) using six hub genes. **(C)** ROC curve analysis of test set (GSE117261) using six hub genes. **(D)** PCA analysis demonstrated the distribution of data sets GSE117261 (red) and GSE24988 (green) before **(upper panel)** and after **(lower panel)** removal of batch effect. The distribution was visualized in scatter plot. **(E)** Expression of six hypoxia induced metabolism associated hub genes in lung tissues of 120 patients with pulmonary hypertension (PH) and 47 control subjects from GSE117261 and GSE24988 after correction of batch effect. Data represent mean ± SEM. *p < 0.05, **p < 0.01, ***p < 0.001 compared to corresponding control subjects, as analyzed by unpaired *t* test or Mann-Whitney *U* test respectively, as appropriate.

As we revealed the six core hypoxia induced metabolism associated hub genes according to cellular metabolomics study and transcriptomics of lung tissues in rodent PH models, we next determined whether these hub genes altered in lung tissues of human PH lungs. To this end, we first examined the expression profile of hub genes in lung tissues of 58 patients with PH and 25 control subjects from GSE117261 data set. In line with the observation from mouse GSE1909 data set, *CAT*, *EPHX1* and *GSTM5* were higher and *GSTO1* was lower in PH patients compared to control subjects (all *p* < 0.05). While *GPX3* was lower in PH patients and *GSTM4* didn’t differ PH from control subjects (Figure 6A). Next, we sought to construct LASSO model to examine whether the metabolism associated hub genes could predict PH. 5 genes were identified with non-zero regression coefficients, and the value of lambda. min was 0.0132,974. The gene-based model index was generated as the following formula: index = CAT*0.157 + GSTM5*0.245 + GPX3*(− 0.288) + GSTM4*(− 0.346) + GSTO1*(− 0.001) + 2.641. ROC curve analysis indicated that area under the curve (AUC) of the 5-gene-based model was 0.90 in the training set from GSE117261 (Figure 6B) and 0.83 in the test set from GSE117261 (Figure 6C), suggesting the established LASSO model might be served as a predictor of PH.

To test the six hypoxia induced metabolism associated hub genes in a larger cohort, we then sought to combine two human PH cohorts (GSE117261 and GSE24988) consisting 120 PH patients and 47 control subjects. We noticed the robust batch effect between these two data sets and used ComBat function in the sva package to merge them and remove the batch effect (Figure 6D). Next, we explored the expression of six hub genes in lung tissues of the merged data sets from GSE117261 and GSE24988 after correction of batch effect. PH patients still exhibited higher *EPHX1* and *GSTM5* and lower *GSTO1* and *GPX3* in lung tissues compared to control subjects (all *p* < 0.05), *CAT* and *GSTM4* didn’t differ from PH and control subjects (Figure 6E).

## Discussion

In the present study our metabolomics analysis at cellular level has identified altered metabolite profiles in rat PASMCs in response to hypoxia and MAGs based on enriched metabolite sets under hypoxia stress. In combination of the transcriptomics data from GEO database, we also figured out differentially expressed MAGs in mouse lungs of hypoxia induced PH. In addition, we identified metabolism associated DEGs in smooth muscle cells of Sugen 5416/hypoxia induced PH rats at a single cell resolution. Our findings highlight the critical function of metabolic adaptations in supporting PASMC proliferation and pulmonary vasculature remodeling under hypoxic condition. We also demonstrated six metabolism associated hub genes and confirmed the alteration of those hub gene expressions in lung tissues of PH patients relative to control subjects, thus providing some insight into the pathogenesis of pulmonary vascular remodeling in PH.

Metabolite perturbation is a very common feature in pulmonary hypertension. In 2014, higher levels of bile acid metabolites in PAH lung tissues were revealed via an untargeted metabolomics method (Zhao et al., 2014). A comprehensive metabolomics study in 2017 identified a distinguishing plasma metabolite pattern in a cohort of well-phenotyped PAH patients from both healthy and symptomatic disease control subjects without PAH, with some of the metabolites of prognostic value in PAH. Changes in the levels of metabolites over time were associated with survival (Rhodes et al., 2017). Circulating indoleamine 2,3-dioxygenase-dependent tryptophan metabolites, tricarboxylic acid intermediates, purine metabolites and arginine-nitric oxide metabolites were revealed to be associated with hemodynamic indicators of right ventricular-pulmonary vascular function by targeted mass spectrometry (Lewis et al., 2016). Our previous study also showed higher plasma levels of spermine in two independent cohorts of idiopathic PAH patients by targeted metabolomic strategy and inhibition of spermine synthesis could inhibit PASMC proliferation *in vitro* and retard PH progression *in vivo* (He et al., 2020). All the findings implicate the disrupted metabolites could serve as potential biomarkers in PH and the correction of metabolism disturbance via modulation of related metabolism associated genes (eg. SMS encoding spermine synthase) might represent a novel therapeutic approach to treat PH.

It is increasingly recognized that the mitochondria, as the orchestrator of energy metabolism, in pulmonary vascular cells exhibit decreased oxidative phosphorylation and increased glycolysis, supporting the existence of the Warburg effect in PH (Cottrill and Chan, 2013; Dabral et al., 2019; Rhodes, 2020). Hypoxia is recognized as a contributor to pulmonary vascular remodeling and can induce local inflammation in pulmonary vasculature by release of growth factors with autocrine/paracrine effects on pulmonary arterial endothelial cells as well as PASMCs (Meuchel et al., 2011; Helan et al., 2014). Several studies have been focused on the imbalanced oxidative phosphorylation/glycolysis and metabolite maladaptation in hypoxic condition in PH. The expression of iron-sulfur scaffold protein BOLA3 (BolA Family Member 3) was reduced in endothelial cells of PH in a hypoxia-dependent manner. BOLA3 deficiency in endothelial cells were demonstrated to activate glycolysis and fatty acid oxidation and drive the production of reactive oxygen species governing proliferative and apoptosis-resistant phenotype of endothelial cells. Endothelial cell knockdown of BOLA3 dysregulate glycine metabolism and promote hemodynamic and histological manifestations of PH, which could be restored via overexpression of pulmonary vascular BOLA3 and glycine supplementation (Yu et al., 2019). Another study showed a metabolic reprogramming towards aerobic glycolysis in PH fibroblasts compared to control fibroblasts. Increased expression of C-terminal Binding Protein-1 (a transcriptional co-repressor that is activated by increased free-NADH secondary to glycolytic reprogramming) was revealed in the lungs of hypoxia-induced experimental PH and in idiopathic PAH patients to orchestrate a network of genes regulating cell proliferation and inflammation. The inhibition of this transcriptional factor was shown to correct the aberrant metabolic, hyper-proliferative and inflammatory phenotype of bovine and human PH fibroblasts and in hypoxic mice (Li et al., 2016). However, the metabolite disturbances in PASMCs under hypoxia stress and their metabolite associated genes in response to hypoxia *in vitro* and in the context of hypoxic pulmonary hypertension *in vivo* remains largely unexplored. Our findings of the metabolite associated genes could provide some novel metabolic targets for diagnostic and therapeutic development in this devastating disease.

In this study, we also identified a list of potential small molecular compounds that might reverse the metabolism associated genes under hypoxia stress. Blebbistatin, ranking the top in the list as a cell-permeable selective inhibitor of myosin II ATP activity, has been reported to reduce neointimal formation and luminal obstruction after vascular injury. It was also evident that blebbistatin had a dose-dependent inhibitory effect on DNA replication and cell proliferative responses to platelet-derived growth factor-BB, a critical trigger in PH progression (Huang et al., 2011). In addition, blebbistatin was shown to inhibit endothelial cell-derived microparticles and platelet-derived microparticles production, thus alleviating ischemia-reperfusion induced pulmonary vascular leakage and lung injury (Zhang et al., 2020). All the findings indicate that blebbistatin might modulate the pulmonary vasculature and open a new avenue for the treatment of PH.

A total of six metabolism associated hub genes (*Cat*, *Ephx1*, *Gpx3*, *Gstm4*, *Gstm5*, and *Gsto1*) were identified. An increase of catalase (hydrogen peroxide catalyser) encoded by *Cat* was demonstrated in both hypoxic mouse lung tissues as well as in lung tissues of PH patients in our study, which was in line with the increased catalase expression in hypoxia PASMCs in an AMPK- and FoxO1-dependent manner in another study (Awad et al., 2014). Additionally, catalase markedly inhibited relaxation mediated by endothelium-dependent hyperpolarization. The subsequent nitrosative stress may be potential triggers of the onset of PH (Tanaka et al., 2018). However, role of catalase in PH is controversial, it has been reported that chronic hypoxia induced reactive oxygen species production could be reversed by catalase (Song et al., 2021). Mice with mitochondrial targeted catalase overexpression attenuated hypoxia-induced PH and proliferative markers (Adesina et al., 2015). Therefore cell-specific *Cat* deletion would be of value to discern the role of catalase in the development of hypoxic PH. The soluble epoxide hydrolase (sEH) encoded by *Ephx2* metabolizes anti-inflammatory epoxyeicosatrienoic acids (EETs) to a less active dihydroxy derivatives. Inhibition of sEH attenuated monocrotaline induced PH in rats. As another key enzyme responsible for EETs hydrolysis *in vivo* (Edin et al., 2018), microsomal epoxide hydrolase (mEH) encoded by *Ephx1* remains to be fully elucidated. Study by Marowsky et al. identified the localization of mEH in brain vascular structure (endothelial and smooth muscle cells). mEH in their study was also reported to have a higher apparent affinity compared to sEH, suggesting that mEH might still contribute to EET turnover even with low substrate (EETs) concentrations (Marowsky et al., 2009). However, the role of *Ephx1* in pulmonary vascular remodeling was unexplored, and whether the two epoxide hydrolases have individual or distinct role in PH warrants further investigation. Recently, Gpx3 has been identified as a novel regulator of insulin receptor expression, and Gpx3 dysregulation would impact insulin receptor and lead to insulin resistance, a not uncommon feature in PAH patients (Pugh et al., 2013; Bradley and Bradley, 2014; Hauffe et al., 2020). In addition, Loss of glutathione peroxidase 3 encoded by *Gpx3* induces ROS and contributes to prostatic hyperplasia (Kim et al., 2020). Patients with systemic sclerosis-related PH displayed lower serum levels of glutathione peroxidase 3 encoded by *Gpx3* (Sun et al., 2020), which was consistent with our observation of a reduction of *Gpx3* in lung tissues of PH patients transcriptionally. However, the role of Gpx3 is worth further scrutiny. *Gstm4* and *Gstm5* encode the proteins belonging to glutathione S-transferases, which are involved in limiting oxidative damage, a critical driver to the development of PH, to tissue (Strange et al., 2001). There is one study showing that glutathione S-transferase mu1 (GSTM1) was significantly decreased in the irreversible CHD-PAH patients, suggesting GSTM1 may be a potential biomarker and target in the irreversibility CHD-PAH (Huang et al., 2018). However, the antioxidant role of Gstm4 and Gstm5 in PH remains unknown. *Gsto1* encode glutathione transferase omega 1, which is a constitutively active deglutathionylating enzyme and required for the lipopolysaccharide-stimulated induction of NADPH oxidase 1 and the production of reactive oxygen species in macrophages (Menon et al., 2014). It also serves as a regulator of the nod-like receptor family, pyrin domain containing 3 (NLRP3) inflammasome via the modulation of NEK7 (Hughes et al., 2019). Of note, NLRP3 inflammasome and its regulated pro-inflammatory cytokines are identified to contribute to pathogenesis of PAH (Tang et al., 2015; Deng et al., 2019). Whether Gsto1 could modulate pulmonary vascular remodeling via NLRP3 or other biological activities remains to be elucidated. Our observation of the alteration of glutathione S-transferases in lung tissues of experimental PH as well as PH patients pinpoints the necessity to determine whether the distinct glutathione S-transferases has separating or overlapping functions in the development of PH, which might provide some insights for the treatment of various diseases triggered by oxidative stress.

There are some limitations in our study. First, in terms of dataset selection, not all datasets for PAH lung tissues were included in our study and our datasets were selected mainly based on sample size, which we considered as a very important factor for data mining. We were also unable to detect the transcriptional expression of hub genes in human lung tissues owing to the lack of transcriptomics studies from human PH due to hypoxia. However, the difference in expression of hub genes from lung tissues of PH patients compared to control subjects underlines their role in the common pathogenesis during the development of PH. Second, the differentially expressed MAGs are underrepresented given that the sample size of hypoxia induced PH mice is limited. Therefore, the lung tissue of hypoxic PH rodent models with larger sample size warranted further investigation and would add more value for the identification of MAGs. In addition, only one single cell RNA sequencing data from PH rodent models is available as of today, hence, hypoxic pulmonary arteries instead of whole lung tissues at the single cell resolution would provide more precise information in the alteration of PASMCs in response to hypoxia, which might aid in the discovery of mechanisms underlying hypoxic PH and thus lead to improved targeted therapies.

## Conclusion

We identified a metabolic profile distinguishing hypoxia-treated from control PASMCs. The combination of the transcriptomics and metabolomics studies allow us to reveal six hypoxia-induced metabolism associated hub genes in response to hypoxia. This would shed some light on the molecular mechanism in hypoxic PH and provide potential therapeutic targets for PH.

## Data Availability

The datasets presented in this study can be found in online repositories. The names of the repository/repositories and accession number(s) can be found in the article/Supplementary Material.

## Abbreviations

ANOVA, Analysis of variance; CMap, Connectivity map; DEGs, Differentially expressed genes; GEO, Gene Expression Omnibus; FC, Fold change; LC/MS, Liquid chromatography-mass spectrometry; LASSO, Least absolute shrinkage and selection operator; MAGs, Metabolism associated genes; PLS-DA, Partial least squares discriminant analysis; PPI, Protein-protein interaction; PH, Pulmonary hypertension; PASMCs, Pulmonary arterial smooth muscle cells; PBS, Phosphate-buffered saline; ROC, Receiver operating characteristic; SuHx, Sugen 5416/hypxoixa; SEM, Standard error of them mean; SphK1, Sphingosine kinases 1; UMAP, Uniform manifold approximation and projection; VIP, Variable important in projection.
