# Corrigendum: Identification of Hypoxia Induced Metabolism Associated Genes in Pulmonary Hypertension

**DOI:** 10.3389/fphar.2021.810178

**Published:** 2021-12-15

**Authors:** Yang-Yang He, Xin-Mei Xie, Hong-Da Zhang, Jue Ye, Selin Gencer, Emiel P. C. van der Vorst, Yvonne Döring, Christian Weber, Xiao-Bin Pang, Zhi-Cheng Jing, Yi Yan, Zhi-Yan Han

**Affiliations:** ^1^ School of Pharmacy, Henan University, Kaifeng, China; ^2^ State Key Laboratory of Cardiovascular Disease and FuWai Hospital, Chinese Academy of Medical Sciences and Peking Union Medical College, Beijing, China; ^3^ Institute for Cardiovascular Prevention (IPEK), Ludwig-Maximilians-University Munich, Munich, Germany; ^4^ DZHK (German Centre for Cardiovascular Research), Partner Site Munich Heart Alliance, Munich, Germany; ^5^ Interdisciplinary Center for Clinical Research (IZKF), RWTH Aachen University, Aachen, Germany; ^6^ Institute for Molecular Cardiovascular Research (IMCAR), RWTH Aachen University, Aachen, Germany; ^7^ Department of Pathology, Cardiovascular Research Institute Maastricht (CARIM), Maastricht University Medical Centre, Maastricht, Netherlands; ^8^ Department of Angiology, Swiss Cardiovascular Center, Inselspital, Bern University Hospital, University of Bern, Bern, Switzerland; ^9^ Department of Biochemistry, Cardiovascular Research Institute Maastricht (CARIM), Maastricht University Medical Centre, Maastricht, Netherlands; ^10^ Munich Cluster for Systems Neurology (SyNergy), Munich, Germany; ^11^ State Key Laboratory of Complex, Severe, and Rare Diseases, and Department of Cardiology, Peking Union Medical College Hospital, Chinese Academy of Medical Sciences and Peking Union Medical College, Beijing, China

**Keywords:** pulmonary hypertension, hypoxia, metabolism associated genes, metabolomics, transcriptomics

In the original article, there was a mistake in the citation as published. In the citation, commas were missing between “He Y-Y and Xie X-M, Xie X-M and Zhang H-D, Zhang H-D and Ye J, Pang X-B and Jing Z-C, Jing Z-C and Yan Y”. The corrected citation should be *“He Y-Y, Xie X-M, Zhang H-D, Ye J, Gencer S, van der Vorst EPC, Döring Y, Weber C, Pang X-B, Jing Z-C, Yan Y and Han Z-Y (2021) Identification of Hypoxia Induced Metabolism Associated Genes in Pulmonary Hypertension. Front. Pharmacol. 12:753727. doi:10.3389/fphar.2021.753727.”*

In addition, there was a mistake in Figure 6 as published. The x-axis labels for Figures 6B and 6C were incorrectly labelled as “specficity”. The corrected Figure 6 appears below.

**FIGURE 6:**
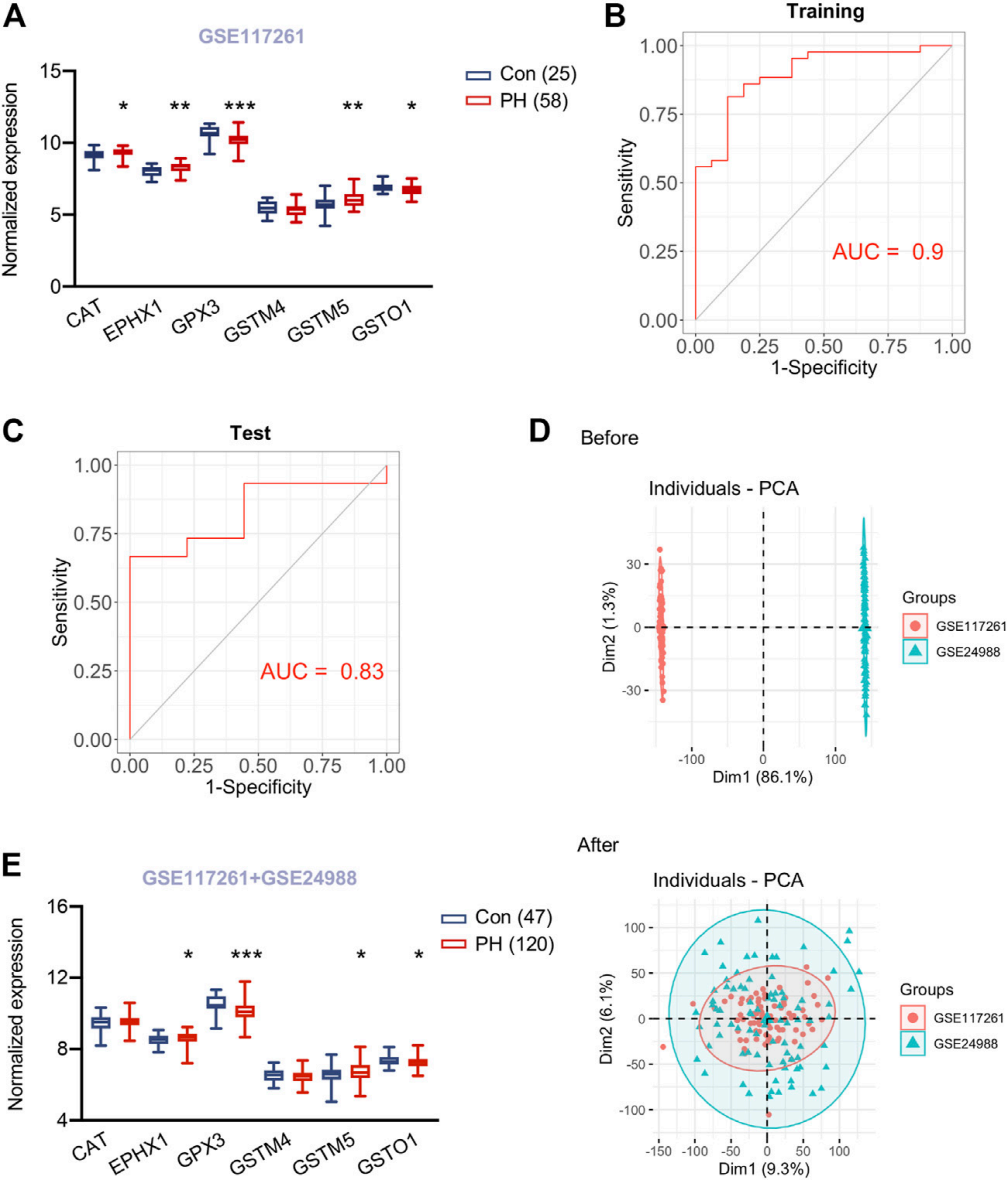
Validation of hub genes in lungs of PH patients and a predicting model for PH. **(A)** Expression of six hypoxia induced metabolism associated hub genes in lung tissues of 58 patients with pulmonary hypertension (PH) and 25 control subjects from GSE117261. **(B)** ROC curve analysis of training set (GSE117261) using six hub genes. **(C)** ROC curve analysis of test set (GSE117261) using six hub genes. **(D)** PCA analysis demonstrated the distribution of data sets GSE117261 (red) and GSE24988 (green) before **(upper panel)** and after **(lower panel)** removal of batch effect. The distribution was visualized in scatter plot. **(E)** Expression of six hypoxia induced metabolism associated hub genes in lung tissues of 120 patients with pulmonary hypertension (PH) and 47 control subjects from GSE117261 and GSE24988 after correction of batch effect. Data represent mean ± SEM. *p < 0.05, **p < 0.01, ***p < 0.001 compared to corresponding control subjects, as analyzed by unpaired *t* test or Mann-Whitney *U* test respectively, as appropriate.

The authors apologize for this error and state that this does not change the scientific conclusions of the article in any way. The original article has been updated.

