# Supplementary material for: Identification of Hypoxia Induced Metabolism Associated Genes in Pulmonary Hypertension
Source: Front Pharmacol. 2021 Nov 5;12:753727. doi: 10.3389/fphar.2021.753727 (PMC8602807; doi:10.3389/fphar.2021.753727)
Supplement: Supplementary file 1 [file DataSheet1.docx]

**Supplementary Materials**

**Identification of** **hypoxia induced metabolism associated genes in pulmonary hypertension**

Yang-Yang He^1^, Xin-Mei Xie^1^, Hong-Da Zhang^2^, Jue Ye^2^, Selin Gencer^3^, Emiel P.C. van der Vorst^3-7^, Yvonne Döring^3,4,8^, Christian Weber^3,4,9,10^, Xiao-Bin Pang^1^, Zhi-Cheng Jing^11^, Yi Yan^3,4,^*, Zhi-Yan Han ^2,^*

1. School of Pharmacy, Henan University, Henan, China;
2. State Key Laboratory of Cardiovascular Disease and FuWai Hospital, Chinese Academy of Medical Sciences and Peking Union Medical College, Beijing, China;
3. Institute for Cardiovascular Prevention (IPEK), Ludwig-Maximilians-University Munich, Munich, Germany;
4. DZHK (German Centre for Cardiovascular Research), partner site Munich Heart Alliance, Munich, Germany;
5. Interdisciplinary Center for Clinical Research (IZKF), RWTH Aachen University, Aachen, Germany;
6. Institute for Molecular Cardiovascular Research (IMCAR), RWTH Aachen University, Aachen, Germany;
7. Department of Pathology, Cardiovascular Research Institute Maastricht (CARIM), Maastricht University Medical Centre, Maastricht, Netherlands
8. Department of Angiology, Swiss Cardiovascular Center, Inselspital, Bern University Hospital, University of Bern, Switzerland;
9. Department of Biochemistry, Cardiovascular Research Institute Maastricht (CARIM), Maastricht University Medical Centre, Maastricht, Netherlands;
10. Munich Cluster for Systems Neurology (SyNergy), Munich, Germany;
11. State Key Laboratory of Complex, Severe, and Rare Diseases, and Department of Cardiology, Peking Union Medical College Hospital, Chinese Academy of Medical Sciences and Peking Union Medical College, Beijing, China.

***Correspondence to:**

Yi Yan, Institute for Cardiovascular Prevention, Ludwig-Maximilians-University Munich, Pettenkoferstreet 8a, 80336 Munich, Germany. E-mail: [yi.yan@med.uni-muenchen.de](file:///Users/yannie/Documents/__/00_Metabolomics_Project%201_rPASMC_Hx/yi.yan@med.uni-muenchen.de)

Zhi-Yan Han, State Key Laboratory of Cardiovascular Disease and FuWai Hospital, Chinese Academy of Medical Sciences and Peking Union Medical College, 167 Beilishi Road, Beijing 100037, China. E-mail: [zhiyanhan2006@hotmail.com](mailto:zhiyanhan2006@hotmail.com)

**Supplementary Table 1. Gradient elution steps for liquid chromatography-mass spectrometry assay**

| Time (min) | Flow rate (mL/min) | Solvent A (%) | Solvent B (%) |
| --- | --- | --- | --- |
| 0 | 0.5 | 95 | 5 |
| 1 | 0.5 | 90 | 10 |
| 8 | 0.5 | 55 | 45 |
| 12 | 0.5 | 45 | 55 |
| 13 | 0.5 | 5 | 95 |
| 15 | 0.5 | 95 | 5 |
| 16 | 0.5 | 95 | 5 |

**Supplementary Table 2. 1259 metabolism associated genes identified in GeneCards**

| Gls | Gatm | Pla2g4a | Uros | Mthfr | Nt5e | Cyp3a5 | Parp10 | Hmgcl | Apoc3 |
| --- | --- | --- | --- | --- | --- | --- | --- | --- | --- |
| Glud1 | Nos1 | Slc7a5 | Urod | Cyp1a2 | Akt1 | Mpo | Parp16 | Mthfd1 | Tlr4 |
| Glul | Nos3 | Atic | Oxt | Cyp2e1 | Ppargc1a | Nr3c1 | Jak2 | Hadh | Mt-cyb |
| Ppat | Pomc | Gch1 | Srsf2 | Cyp2d6 | Nqo1 | Gstp1 | Gstt1 | Mttp | Agxt |
| Nadsyn1 | Pnpo | Slc3a2 | Phgdh | Slc6a3 | Ppig | Akr1a1 | Drd1 | Hbb | Tpo |
| Pfas | Ren | Ifng | Hsd3b2 | Cyp1a1 | Hprt1 | Nadk2 | Parp4 | Mthfd1l | Aass |
| Gmps | Gh1 | Por | Tyrp1 | Aldh2 | Xdh | Ldha | Parp9 | Csf2ra | Cd36 |
| Gls2 | Ins | Tyms | Dhcr7 | Sirt1 | Adh1b | Akr1b1 | Ptgis | Calca | Nme1 |
| Dglucy | Prodh | Mthfs | B4galt1 | Lep | Cyp2c8 | Nmnat1 | Nadk | Kcnj11 | Hadhb |
| Glud2 | Cyp17a1 | Alox5 | Srsf3 | Cyp3a4 | Fgf23 | Nmnat2 | Mmp9 | Pfkm | Nat2 |
| Slc16a10 | Cyp11b2 | Th | Slc7a1 | Comt | Crp | Nmnat3 | Idh1 | Irs1 | Ar |
| Cad | Hmbs | Sst | Pign | Parp1 | Gck | Chrna7 | Fmo1 | Ucp2 | Gk |
| Gfpt1 | Cyp19a1 | Sod1 | Gnrh1 | Aox1 | Il10 | Nmrk1 | Tgfb1 | Casr | Cs |
| Grm1 | Hmgcr | Tdo2 | B4galt7 | Maoa | Nt5c1a | Naprt | Mapt | Lmna | Acox1 |
| Kyat1 | Crh | Avpr2 | Nsdhl | Cd38 | Gsr | Nnmt | Naxd | Abcc8 | Prps1 |
| Asns | Igf1 | Slc25a13 | Ndufaf5 | Adipoq | Dbh | Nmrk2 | Gldc | Suox | Sdhb |
| Grm3 | Cyp11b1 | Arg2 | Acta2 | Pnp | Aldh9a1 | Nudt12 | Mtr | Gba | Ttr |
| Grm2 | Alas2 | Nos2 | Srpk1 | Pparg | Ephx1 | Sirt3 | Upb1 | Lipc | Idua |
| Ctps1 | Alb | Prmt1 | Slc1a4 | Fmo3 | Gcg | Nnt | Mtrr | Ampd1 | Star |
| Ctps2 | Cyp21a2 | Prmt5 | Edn1 | Drd2 | Insr | Qprt | Aldh7a1 | Cpt1a | Gla |
| Gatc | Qdpr | Carm1 | Srsf5 | Cyp2c9 | Serpine1 | Oprm1 | Alpl | Pth | Aprt |
| Gfpt2 | Ptgs2 | Aldh18a1 | Srsf7 | Il6 | Gpt | Lpa | Apoe | Galk1 | Sdha |
| Qrsl1 | Pmm2 | Azin2 | Nppb | Cnr1 | Dhfr | Hcar2 | Apob | Cdkn2a | Atp7b |
| Gatb | Fech | Prmt7 | Pgap2 | Kynu | Vdr | Bst1 | Cp | Ampd3 | Gusb |
| Grm5 | Lbr | Prmt6 | Cfap47 | Cyp2c19 | Bglap | Sirt2 | Pten | Polg | Kcnj1 |
| Ass1 | Hadha | Slc6a8 | Ptgs1 | Enpp1 | Cbs | Enpp3 | G6pc | Cyp11a1 | Slc4a1 |
| Asl | Prl | Avpr1a | Abcb11 | Lpl | Nt5c2 | Serpina3 | Gcdh | Nme2 | Shbg |
| Otc | Tnf | Aqp2 | Ddah2 | Apoa1 | Ldlr | Chat | Mmut | Tpi1 | Akt2 |
| Cps1 | F9 | Alg13 | Fdft1 | Ccl2 | Pon1 | Cyp2a13 | Tpk1 | Psap | Pck1 |
| Arg1 | Adsl | Gpaa1 | Manf | Ppara | Tf | Sirt6 | Galt | Cyp1b1 | Fxn |
| Gamt | Mvk | Odc1 | Slc25a38 | Hif1a | Slc2a1 | Sirt4 | Sftpc | Atp7a | Tymp |
| Slc7a7 | Btd | Slc3a1 | Psat1 | Gstm1 | Abat | Nt5c3a | Pik3ca | Fh | Dnmt1 |
| Slc25a15 | Il1b | Pigt | Oca2 | Cat | Hmox1 | Ache | Dld | Smpd1 | Igf1r |
| Avp | Tp53 | Prmt3 | Ckmt1b | Ugt1a1 | Npy | Mapk1 | Hfe | Slc17a5 | Cftr |
| Nags | G6pd | Piga | Kng1 | App | Ndufs4 | Lepr | Fah | Maob | Mt-nd1 |
| Oat | Serpina1 | Pigs | Alas1 | Nr3c2 | Ogdh | Nt5c1b | Tat | Mat1a | Fasn |
| Cpox | Acadm | Srsf1 | Nampt | Bdnf | Hnf4a | Sirt7 | Lcat | Gnas | Aldob |
| Pigv | Nppa | Ppox | Cyp2a6 | Ghrl | Ace | Nt5c | Acadvl | Nr1h2 | Ugt1a6 |
| Pah | Dpyd | Slc7a2 | Cyp2b6 | Hsd11b2 | Ada | Sirt5 | Cpt2 | Hk1 | Mt-atp6 |
| Tyr | Hlcs | Alad | Ggt1 | Gapdh | H6pd | Nt5m | Pc | Ahcy | Ryr1 |

| **Supplementary Table 2 Continued: 1259 metabolism associated genes identified in GeneCards** | | | | | | | | | |
| --- | --- | --- | --- | --- | --- | --- | --- | --- | --- |
| Prkaa1 | Mir140 | Spr | Aldh1b1 | Slc30a8 | Slc22a12 | Lacc1 | Tnfrsf11a | Prkaca | Crat |
| Aldoa | Fhit | Cndp1 | Kl | Csf2rb | Slc2a9 | Pcca | Fpgs | Slc28a3 | Fto |
| Igf2r | Hykk | Nr1h4 | Plg | Ide | Mc3r | Mocs1 | Msmb | Nr1i2 | Pura |
| Ctsd | Nit2 | Cfh | Slc1a3 | Tfrc | Paics | Etfdh | Slc28a1 | Tmsb15a | Glyctk |
| Dpys | Nans | Egfr | Aptx | Dph1 | Sftpb | Ngf | Acadsb | Agt | Pgk1 |
| Acaca | Kit | Ethe1 | Cox5a | Bckdk | Vkorc1 | Dclre1c | Acad8 | Slc25a19 | Taz |
| Pik3c2a | Hao2 | Htr1a | Cblif | Aldh3b1 | Dguok | Mmab | Mpv17 | Gmpr | Slc29a1 |
| Gss | Slc5a10 | Nts | Irs2 | Kcnh2 | Adss2 | Pde5a | Guk1 | Hras | Braf |
| Cycs | Hpd | Galc | Pdhx | Dlst | Umod | Prkaa2 | Pklr | Tnfsf11 | En1 |
| Mtor | Ca2 | Minpp1 | Sult1a1 | Cyb5a | Fbp1 | Cyp27b1 | Eif2ak3 | Plin1 | Mt-nd6 |
| Esr1 | Or2t11 | Ghr | Bckdha | Ido1 | Ampd2 | Nhej1 | Ivd | Atp5f1a | Cd79a |
| Slc12a3 | F2 | Arsa | Taldo1 | Ftl | Adss1 | Slc17a1 | Ucp3 | Aldh16a1 | Gsta1 |
| Mt-co1 | Pcsk9 | Gys1 | Pla2g6 | Abhd5 | Slc2a2 | Abca1 | Slc22a6 | Slc4a8 | Gclc |
| Cav1 | Mb | Slc40a1 | Mc4r | Nbpf3 | Cxcl8 | Impdh1 | Etfb | Polg2 | Gpx4 |
| Tpmt | Bche | Bckdhb | Hpr | Cog2 | Slc37a4 | Pnpp1 | Mpg | Nudt1 | Gpx3 |
| Hexb | Hamp | Sts | Slc18a2 | Slc16a1 | Acads | Slc4a4 | P2rx7 | Flad1 | Mgst1 |
| Prkn | Ca5a | Carnmt1 | Pvalb | Slc5a1 | Mtap | Pccb | Alms1 | Dpyd-it1 | Gsto1 |
| Ephx2 | Gast | Gstz1 | Hnf1a | Cacna1s | Retn | Slc28a2 | Naglu | Oplah | Mgst3 |
| Egf | Nkx2-1 | Htr2a | Slc12a1 | Surf1 | Zap70 | Pkm | Gpx1 | Ak4 | Gsta2 |
| Myc | Cetp | Tph1 | Chga | Msmo1 | Pygm | Phkg2 | Aldh6a1 | Bcs1l | Gstm3 |
| Abcb1 | Hgd | Vip | Col6a2 | Fth1 | Prtfdc1 | Myd88 | Shld2 | Pdzk1 | Gsta4 |
| Adora2a | Apoa4 | Hba1 | Hint2 | Bhmt | Acat1 | Adrb3 | Rrm1 | Sprr2f | Mgst2 |
| Ahr | Hnmt | Pnpla2 | Rbp4 | Alox12 | Rag1 | Tlr2 | Tkt | Prdx1 | Gstm2 |
| Prkab1 | Snca | Aldh3a1 | Krt18 | Grhpr | Pygl | Cyp7a1 | Hsd17b10 | S100a9 | Gsto2 |
| Sod2 | Aldh3a2 | Hexa | Pnpla3 | Ak2 | Ercc6 | Cd40lg | Hibch | Slc22a8 | Ggt7 |
| Hal | Carns1 | Abcc1 | Ttpa | Mocos | Pck2 | Ak1 | Cyp27a1 | Atp5f1d | Ggt5 |
| Uroc1 | Got2 | Fads1 | Actb | Umps | Slco1b1 | Ucp1 | Mefv | Cyp24a1 | Ltc4s |
| Hdc | Lipa | Slc6a4 | Hla-b | Adk | Slc7a9 | Gphn | Fga | Slc29a2 | Gstm4 |
| Hrg | Tp73 | Bcl2 | Sco2 | Itpa | Shmt2 | Etfa | Cd4 | Slc46a1 | Hba2 |
| Ftcd | Lipe | Ttf2 | Syp | Hoga1 | Il18 | Agl | Hsp90aa1 | Pde4a | Ggt6 |
| Dmgdh | Naga | Ehhadh | Uqcrfs1 | Galns | Abcc4 | Pgm1 | Acadl | Abcc2 | Gsta3 |
| Gcsh | Tfr2 | Slc11a2 | Slc39a14 | Dck | B2m | Acad9 | Pdha1 | Ogg1 | Gstm5 |
| Ddc | Hcn1 | Lct | Pcbd1 | Gda | Rrm2b | Gaa | Slc19a1 | Stk11 | Mmadhc |
| Lmbrd1 | Pdxk | Glb1 | F12 | Abca3 | Tnfrsf11b | Mocs2 | Apoa2 | Tsc2 | Tcf7l2 |
| Aldh4a1 | Rb1 | Scd | Aspa | Prps2 | Shmt1 | Muc1 | Il1r1 | Ifnb1 | Gpx2 |
| Cubn | Hars1 | Ca1 | Htr3a | Nlrp3 | Phka2 | Npc1 | Dpp4 | Pik3r1 | Gstk1 |
| Mmachc | Hint1 | Aoc1 | Dph5 | Prps1l1 | Impdh2 | Cldn16 | Adora3 | L2hgdh | Gstt2 |
| Plpbp | Cck | Amdhd1 | Scarb1 | Dars2 | Ugt1a8 | Pnpt1 | Sars2 | Brca1 | Ggt2 |
| Mir21 | Cth | Hk2 | Grp | Gart | Slc2a4 | Hnf1b | Cdk1 | Adcy10 | Gsta5 |
| Kcnq2 | Pts | Clcnkb | Ptpn1 | Nudt15 | Abcg2 | Cdk2 | Agpat2 | Nfe2l2 | Hpgds |

**Supplementary Table 2 Continued: 1259 metabolism associated genes identified in GeneCards**

| Gstt2b | Igfbp3 | Mir31 | Nat8 | Pgd | Slc7a11 | Ndufaf3 | Gale | Ano5 | Pomk |
| --- | --- | --- | --- | --- | --- | --- | --- | --- | --- |
| Glo1 | H2ac18 | Mapk14 | Npc2 | Clu | Ces1 | Cacna1a | Slpi | Fbp2 | Man2a1 |
| Gpx5 | Hp | Mir222 | Atf4 | Bax | Plod3 | Slc25a20 | Uqcrc2 | Man1b1 | Tardbp |
| Hagh | Cdh1 | Stat3 | Mir10b | Adh1c | Mir205 | Setx | Slc25a42 | Dpm3 | Fktn |
| Mir17 | Vegfa | Mir143 | Ppard | Socs3 | Nat1 | Mmd | Wt1 | Slc2a5 | Il1rn |
| Bmp6 | Selp | Tert | Mir191 | Fn1 | Btnl2 | Smn2 | Ndufa6 | Mogs | Man2c1 |
| Srebf1 | Gpx8 | Txn | Il2 | Txndc12 | Mir199a1 | Aco1 | Slc25a11 | Pfkfb3 | Akr1b10 |
| Gclm | Chac1 | Gstt4 | Hspa4 | Akr1c3 | Hgf | Atxn2 | Tufm | Slc35c1 | Icam1 |
| Fgfr1 | Igfbp1 | Foxo1 | Glrx2 | Smarca4 | Pthlh | Smn1 | Dao | Pfkl | Gm2a |
| Ctnnb1 | Mir125a | Mcee | Secisbp2 | Prkca | Mir15a | Baat | Tmem70 | Sord | Asah2 |
| Gpx7 | Cyb5r3 | Si | Erbb4 | Nfkbia | Xiap | Fmr1 | Lipt2 | Gmppb | Sgsh |
| Ggtlc2 | Brca2 | Mir145 | Kdm4c | Map2k1 | Adrb2 | Cryaa | C4a | Aldoc | Acer3 |
| Mir34a | Selenbp1 | Mmp2 | Anxa5 | Ctns | Cyp4f2 | Ireb2 | Atp8b1 | Pfkfb2 | Degs1 |
| Bmp2 | Mir223 | Mir210 | Calr | Gsk3b | Fgf2 | Mccc1 | Retreg1 | Pfkfb1 | Ugcg |
| Gpx6 | Apoa5 | Tusc3 | Mir203a | Mir23b | Itgb3 | Cfp | Cd55 | Alg14 | Mcoln1 |
| Ptges | Fas | Mir106b | Ikbkb | Dnmt3b | Abcd1 | Serping1 | Mgam | Gckr | Arsb |
| Adh5 | Pdgfrb | Mir25 | Mir16-1 | Mirlet7g | Dyrk1b | Vwf | Pepd | Pfkfb4 | Sumf1 |
| Stat1 | Chka | Mir200c | Prkag2 | Ezh2 | Kdr | Cfhr5 | Csn1s1 | Man2b1 | Sptlc2 |
| Sqstm1 | Agk | Pik3cg | Mir99a | H19 | Lipt1 | Il4 | Lrpprc | Pfkp | Arsh |
| Cdkn3 | Mir29a | Mir23a | Sp1 | Rela | Limk1 | Cfhr4 | Fgf21 | Gmds | Smpd2 |
| Mir181a1 | Cdk4 | Hdac1 | Msh2 | Hspa5 | Akr1c2 | Sftpd | Eln | Tigar | Lamp2 |
| Casp3 | Spp1 | Src | Mir195 | Slc25a4 | Nr5a1 | Foxp3 | Cyp26a1 | Gpi | Gba2 |
| Ccnd1 | Mirlet7d | Nfkb1 | Cdkn1a | Arnt | Ctsb | Mccc2 | Sptlc1 | Alg2 | Scarb2 |
| Mir146a | Pgr | Ep300 | Faslg | Raf1 | C3 | Slc25a12 | Cfhr2 | Pmm1 | Sgpp1 |
| Atm | Trpv6 | Echs1 | Mme | Plat | Serac1 | Mecp2 | Mbl2 | Pomgnt1 | Ugt8 |
| Casp8 | Nbn | Srd5a1 | Abcc6 | Ncor1 | Thbd | Gnmt | Invs | M6pr | Sgpl1 |
| Hsd11b1 | Ggtlc3 | Pdgfb | Mlxipl | Gja1 | Cfi | Asah1 | Pla2g2a | Hk3 | Degs2 |
| Mmp1 | Fgfr2 | Chek2 | Auh | Abcc3 | Adamts13 | F3 | Cst3 | Pomgnt2 | Ctsa |
| Jun | Met | Foxo3 | Ggct | Ccl5 | Cfb | Il17a | Ntrk1 | Hkdc1 | Ids |
| Mir22 | Tp63 | Anpep | Prdx5 | Klf6 | Cd46 | Timp1 | Pank2 | Gmppa | Smpd3 |
| Mapk8 | Apc | Mirlet7c | Mir122 | Rps6kb1 | Iscu | C1s | Pank1 | Gfus | Cers1 |
| Prdx6 | Fgfr4 | Dpep1 | Esd | Ggtlc1 | Mmaa | Ocrl | Pank3 | Mgat2 | Abcg8 |
| Mir27a | Idh2 | Esr2 | Igfbp2 | Ndufs1 | Dgke | Lias | Coasy | Pomt1 | Clcn7 |
| Kras | Slc22a5 | Mir214 | Abcd4 | Nr1i3 | Cfhr1 | Fan1 | Pank4 | Lrp2 | Scp2 |
| Ggt3p | Mir127 | Aldh5a1 | Lox | Runx2 | Abcb7 | Itgam | Ppcs | Tnfrsf1a | Fa2h |
| Erbb2 | Msr1 | Amacr | Ddit3 | Rhoa | Elp1 | Nfu1 | Ppcdc | Nod2 | Sphk1 |
| Mir181b1 | Mir221 | Mapk3 | Slc15a2 | Crebbp | Cfhr3 | Angptl3 | Acss2 | Tkfc | Sptlc3 |
| Igf2 | Fos | Mif | Casp9 | Scap | Ntrk2 | Vtn | Vnn1 | Pomt2 | Acer2 |
| Glrx | Mir20a | Mir200b | Alox5ap | Prlr | Ctla4 | Lpin1 | Mpi | F5 | Sumf2 |
| Chac2 | Srd5a2 | Mir126 | Shc1 | Hspb1 | Tango2 | Tcn2 | Khk | Fkrp | Ormdl3 |

**Supplementary Table 2 Continued: 1259 metabolism associated genes identified in GeneCards**

| Pkd2 | Neu1 | Cers6 | Adprh | Cdh23 | Acp5 | Gfap | Trpv4 | Npb | Trpv1 |
| --- | --- | --- | --- | --- | --- | --- | --- | --- | --- |
| Acer1 | Cerk | Ercc2 | Sphk2 | Becn1 | Atp13a2 | Ccl18 | Hhex | Plp1 | Vapb |
| Sptssa | Gal3st1 | Sgms1 | B4galt6 | Gba3 | Ldlrap1 | Elovl1 | St8sia1 | Pikfyve | Mrap |
| Chit1 | Lamp1 | Sgms2 | Cers2 | Gns | Pink1 | B4galnt1 | Lrrk2 | Rab8a | Ppt1 |
| Tecr | Kdsr | Cers4 | Sgpp2 | Ormdl1 | Trpm6 | Mbp | Plpp3 | Plcb1 | Mapk10 |
| Cers3 | Cers5 | Tfeb | Prdm10 | Plpp1 | Ormdl2 | Sptssb | Oga | Alox12b |  |

**Supplementary Table 3. Differentially expressed genes in smooth muscle cells of lung tissues from SuHx rats compared to controls**

| **Gene symbol** | **P value** | **logFoldChange** |
| --- | --- | --- |
| *Crip1* | 8.79E-05 | 1.90578921 |
| *Tmem30a* | 4.38E-02 | 1.7239028 |
| *S100a4* | 1.25E-03 | 1.69622594 |
| *Ces1d* | 3.59E-03 | 1.67938701 |
| *Mgp* | 3.52E-05 | 1.57962618 |
| *Sspn* | 3.19E-02 | 1.55010024 |
| *AABR07002768.1* | 4.25E-02 | 1.47541474 |
| *Taldo1* | 3.74E-02 | 1.45839086 |
| *Zc3h13* | 2.36E-02 | 1.38755012 |
| *Id3* | 2.22E-02 | 1.37703012 |
| *Ppp6r1* | 3.87E-02 | 1.33929079 |
| *Mbnl2* | 4.03E-02 | 1.32248864 |
| *Idi1* | 3.73E-02 | 1.25725435 |
| *Igfbp7* | 1.11E-02 | 1.2087494 |
| *Ddx55* | 4.87E-02 | 1.18682239 |
| *Supt4h1* | 4.56E-02 | 1.16628225 |
| *Stub1* | 4.44E-02 | 1.15828983 |
| *Acox1* | 3.97E-02 | 1.13883287 |
| *Ctr9* | 3.80E-02 | 1.12405368 |
| *BC028528* | 4.60E-02 | 1.07759393 |
| *Aldh6a1* | 4.33E-02 | 1.06160153 |
| *Cwc15* | 4.24E-02 | 1.02787243 |
| *Pdia3* | 3.61E-02 | 1.00084116 |
| *Mcam* | 4.40E-02 | 0.29389788 |
| *Ltbp1* | 3.19E-02 | -0.5519551 |
| *Gpx3* | 1.81E-02 | -1.1665254 |
| *Mmp2* | 3.67E-02 | -1.5494111 |
